# Local and distant trauma after hypervelocity ballistic impact to the pig hind limb

**DOI:** 10.1186/s40064-016-3160-y

**Published:** 2016-09-07

**Authors:** Jin Chen, Bo Zhang, Wei Chen, Jian-yi Kang, Kui-jun Chen, Ai-min Wang, Jian-min Wang

**Affiliations:** 1 6th Department of Research Institute of Surgery, Daping Hospital, Third Military Medical University, Chongqing, 400042 China; 2 Department of Orthopedics, Daping Hospital, The Third Military Medical University, Chongqing, China; 3 State Key Laboratory of Trauma, Burns and Combined Injury, Daping Hospital, Third Military Medical University, Chongqing, China; 4 Department of Orthopedics, Nanchong Central Hospital, North Sichuan Medical College, Nanchong, China

**Keywords:** Projectile, Hypervelocity, Ballistic impact, Transient cavity, Distant injury

## Abstract

The development of high-energy weapons could increase the velocity of projectiles to well over 1000 m/s. The nature of the injuries caused by the ballistic impact of projectiles at velocities much faster than 1000 m/s is unclear. This study characterizes the mechanical and biochemical alterations caused by high-speed ballistic impact generated by spherical steel ball to the hind limbs of the pig. That the local and distal injuries caused by hypervelocity ballistic impact to the living body are also identified. It is showed that the severity of the injury was positively correlated with the velocity of the projectile. And 4000 m/s seems to be the critical velocity for the 5.6 mm spherical steel ball, which would cause severe damage to either local or distal organs, as below that speed the projectile penetrated the body while above that speed it caused severe damage to the body. In addition, vaporization prevented the projectile from penetrating the body and the consequent pressure wave seems to be the causal factor for the distant damage.

## Background

Penetrating trauma, particularly high-energy injuries, are likely to increase in the future as military assault weapons are increasingly infiltrating the civilian sector via the illegal narcotics trade, and terrorist bombings are becoming more common-place (Petersen and Waterman 2011). Over the past century, the mechanisms by which ballistic impact injure living tissue have been extensively studied (Fackler 1996). At present, there are two mechanisms for local tissue damage after ballistic penetrating injury. One of the mechanisms is permanent cavitation, in which the affected tissue is crushed and destroyed. The second is temporary cavitation, which is caused by temporary cavity pressure generated while projectiles are penetrating the tissue. Temporary cavity pressure could aggravate the injury by oscillating the tissue with high-frequency pressure waves (Fackler 1996; Maiden 2009).

Not only the local tissue but also the distant tissues from the direct injury site are injured after ballistic impact to the torso. Ballistic injury can be roughly divided into three types, surrounding tissue injury, adjacent organ injury and distant organ injury. The surrounding tissue injury is a kind of injury which is surrounding the wounding tract, and with the primary wounding tract there is a direct anatomical link. Adjacent organ injury is a kind of injury which is the adjacent tissues and organs injury by pressure wave directly, usually performed for varying degrees of rupture, bleeding, hematoma, fracture and so on (Kiser et al. 2013). Distant injury, also called as remote effect, is a kind of injury which is foar from wounding tract, and with the primary wounding tract there is no anatomical link. It was reported that the brain is injured after penetrating injury to the extremities or thoraces of dogs. This phenomenon is called the distant or remote effect (Suneson et al. 1990). The primary mechanism of remote injuries remains under investigation, although it is thought that shock waves generated by high velocity projectiles are perhaps the major mechanism of cellular damage to distant tissues, including the nervous system (Maiden 2009).

The severity of a ballistic wound depends on several groups of factors. The first is the characteristics of the projectiles. A shape that causes the projectile to deform, fragment, or change in orientation would expand the extent of the wound. The second group includes the characteristics of the wounded tissue, including the tissue thickness, density and elasticity. Near-water-density, less elastic tissue (e.g., brain, liver, or spleen) may be damaged more severely than more elastic tissue (e.g., muscle) when penetrated by the same projectiles. The third group of factors involves the velocity of the projectiles. Slower projectiles typically disrupt more tissue and cause smaller temporary cavities; however, faster projectiles typically form larger temporary cavities but smaller permanent cavities (Fackler 1996; Hollerman et al. 1990).

Clearly, the extent of a ballistic injury is affected by many factors. However, when considering identical projectiles, according to the Newtonian principle E = 1/2 mv^2^, velocity is a key factor that influences the extent of injury. Until now, ballistic projectile injury and its mechanisms have been studies mainly at speeds of less than 2000 m/s. In the future, with the utilization of high-explosives and the improvement of guns, the projectiles will impact their targets at 2000 m/s and higher velocities. What is it like when projectiles impact living tissue at speeds in excess of 2000 m/s? Are there velocity ranges within which projectiles would cause the same type of injuries? Are the local and distant injuries highly correlated with the velocity when the same projectile is used?

To elucidate these problems, we used spherical metal balls as the projectiles. The right hind limbs of white pigs were injured with projectiles at four velocities (1000, 2000, 3000, and 4000 m/s). The pathological effects on different organs were examined. To compare the injuries to the central nervous system (CNS), we assessed the serum levels of myelin basic protein (MBP), neuron-specific enolase (NSE), and the glial cytoplasmic protein S-100B. Interleukin 6 (IL-6) was also measured to indicate the extent of inflammation. To investigate the biomechanical characteristics of the pressure waves generated by the impacts, we measured the pressure in the vicinity of the site of injury and in the common carotid artery. To assess the size of the temporary cavity, we used a special soap as a target.

## Methods

### Subject and groups

This study was approved by the Science and Technological Committee and the Animal Use and Care Committee of the Third Military Medical University. Twenty-five healthy, young adult white pigs that weighed 42.10 ± 5.51 kg (ranging from 31.9 to 53.3 kg) were used in these studies. All of the animals were accommodated in an accredited animal facility for at least 3 days before the experiments were performed and were fed a standard diet with free access to tap water. The ambient room temperature was maintained between 20 and 25 °C with a 12-h light/dark cycle. Twenty-five animals were randomly divided five groups of five pigs each for four projectile velocity groups (1000, 2000, 3000, and 4000 m/s), which were injured with live ammunition, whereas the fifth group served as the negative controls.

### Animal management

The experimental setting was similar to that reported by Drobin (Drobin et al. 2007). The pigs were anesthetized with intravenous injections of 3 % somnopentyl (30 mg/kg; Merck, Darmstadt, Germany) through the marginal vein of the ear. After administration of anesthetic, the animals were placed supine on a standard operating table. The right femoral artery was dissected and cannulated with a three-way tube for blood sampling. The incision was sutured, and the collection tube was anchored. Heparin (2.5 %) was used as an anti-thrombotic agent in the tube.

After completing the catheterization, the sedated animals were transported for the experiment. The animals were kept warm during the experiments.

### Biomechanics recording system

One pressure transducer (CYG4-1000, Qinming Electronics Co., Ltd., Shanxi, China) was inserted into the left buttock at 11–13 cm from the target point, approximately 2 cm deep and was fastened with thread with the face of the receptor vertically apposed to the skin. Another pressure transducer (ZT-500, Decheng Electronics Co., Ltd., Shanxi, China) was inserted into the left common carotid artery from the level of thyroid cartilage and 4 cm deep, and the face of the receptor was oriented toward the heart.

Signals were acquired using a PXI6133 (National Instruments Inc., Austin, Texas, USA) and were analyzed with Flexpro6.0. Before the experiment, the transducers were calibrated with the aid of an internal calibration system.

### Vulneration

The projectile was a spherical full-metal ball with a diameter of 5.6 mm and a weight of 0.72 g. The target point was located outside of the left hind limb where the muscle is abundant, and 5–6 cm from the middle of the thigh bone.

The anesthetized animals were placed on a special platform in a laterally recumbent position, and the left hind limb was suspended. A two-stage light-gas gun was used to achieve projectile velocities faster than 2000 m/s. A 5.8 mm ballistic rifle equipped with a laser aiming device (Mingguang Instrument Factory, Chongqing, China) was used to achieve a projectile velocity of 1000 m/s. The distance between the muzzle and the target point was fixed at 4 m. Projectile velocities near 1000 m/s were measured with an optical shutter device (B570, AVL List GmbH, Graz, Austria). Projectile velocities higher than 2000 m/s were measured with a laser device (CDKP-OPFIB50-25 laser velocimeter, Shuyou Technology Co., Ltd., Chengdu, China).

Special soap targets (27 cm × 27 cm × 27 cm; weight 27 kg) were shot with projectiles at three different velocities (1000, 2000, and 4000 m/s). The soap block (Wanshun chemical factory, Baoding, Hebei) was mainly composed of (C17H35COO)Na with (C17H35COO)Na/H_2_O weight ratio of 45/55.

During the course of each shot, a high-speed video camera (Photron Fastcam SA5, Photron USA, Inc. San Diego, CA, USA) was used to record the detailed course of injury. The diameter of the largest transient cavity was calculated from the pictures captured by the high-speed camera, and the diameter of middle tibia was used as the scale.

After injury, the entrance and exit defects were examined and their diameters were measured.

### Sample preparation

Blood samples (5 ml) were acquired before each shot and again 30 min, 1, 3, and 6 h after the shot. The samples were centrifuged at 1500*g* for 10 min to obtain serum. The sera were then stored at −70 °C for analysis of MBP, NSE, S-100B and IL-6 expression.

After 6 h of observation, all of the animals were deeply anesthetized and sacrificed by bleeding from the aorta. All of the organs were carefully examined. Samples from the wound tract, lung, liver, kidneys and brain were cut and fixed in 4 % paraformaldehyde (Bosde, Wuhan, China) at 4 °C for morphological evaluation.

### Immunochemical analysis

The cytosolic protein NSE is used as an indicator of neural damage, S-100 is mainly present in the cytosol of astrocytes, whereas MBP is a marker of the myelin sheath (Wang et al. 2004; Saljo et al. 2003). The levels of these three specific proteins in the serum and CSF were determined using an enzyme-linked immunosorbent assay kit (Rapidbio Inc., San Diego, California, USA). The OD value was measured at 450 nm on a plate reader (Multiskan MK3, Thermo Labsystems (Shanghai) Co., Ltd., Shanghai, China).

### Pathological examination

For light microscopy (IX 50; Olympus, Tokyo, Japan), paraffin-embedded samples were sectioned at 1–3 μm in thickness (Yongsheng, Shanghai, China). These samples were stained with hematoxylin-eosin (Chemical Reagent Factory, Shanghai, China).

### Statistics

Values are expressed as the mean ± standard error (SE). Repeated measures and multivariate analysis of variance (RM-ANOVA) processes of the general linear model in SPSS (Version 13.0 for windows, SPSS, Inc., Chicago, IL) were used to explore the differences between and within groups. One-way ANOVA in SPSS was applied to the variation in expression levels of biomarkers. When equal variances were assumed, the method of least significant difference (LSD) was applied. If not, Tamhane’s T2 was applied. In figures, the means are depicted inside 95 % confidence intervals, and *p* values of 0.05 or less are considered significant.

## Results

### General pathology of wound tract

All of the projectiles penetrated the hind limb; however, at a velocity of 4000 m/s, there were no exit wounds in the injured limbs. With the increasing of velocity, the entrance wound became larger (Table 1).

**Table 1:** Features of wound tracts caused by projectiles with different super-high velocities (n = 5)

Group (m/s)	Average velocity (m/s)	Area of entrance^△^ (cm^2^)	Area of exit^△^ (cm^2^)	Palor area^△^ (cm^2^)	Range of contusion^※^ (cm)
1000	998.40 ± 6.79	0.69 ± 0.08	0.22 ± 0.02	7.20 ± 1.36	0.90 ± 0.12
2000	2046.00 ± 69.83	24.70 ± 4.32	3.35 ± 0.71 a	147.4 ± 11.65 b	2.74 ± 0.11 a
3000	2962.00 ± 28.04	124.50 ± 8.37 a	5.50 ± 0.87 a	260.80 ± 14.84 a	3.72 ± 0.25 a
4000	3991.6 ± 35.87	257.2 ± 32.15 a	^●^	516.40 ± 67.87 a	4.80 ± 0.25 a

^△^The area was acquired from the product of the longest lateral axis and the vertical axis of the injured skin

^※^This range was calculated according to the color

^●^There was no exit. Compared the group of 1000 m/s, a < 0.01, b < 0.05

**Fig. 1:**
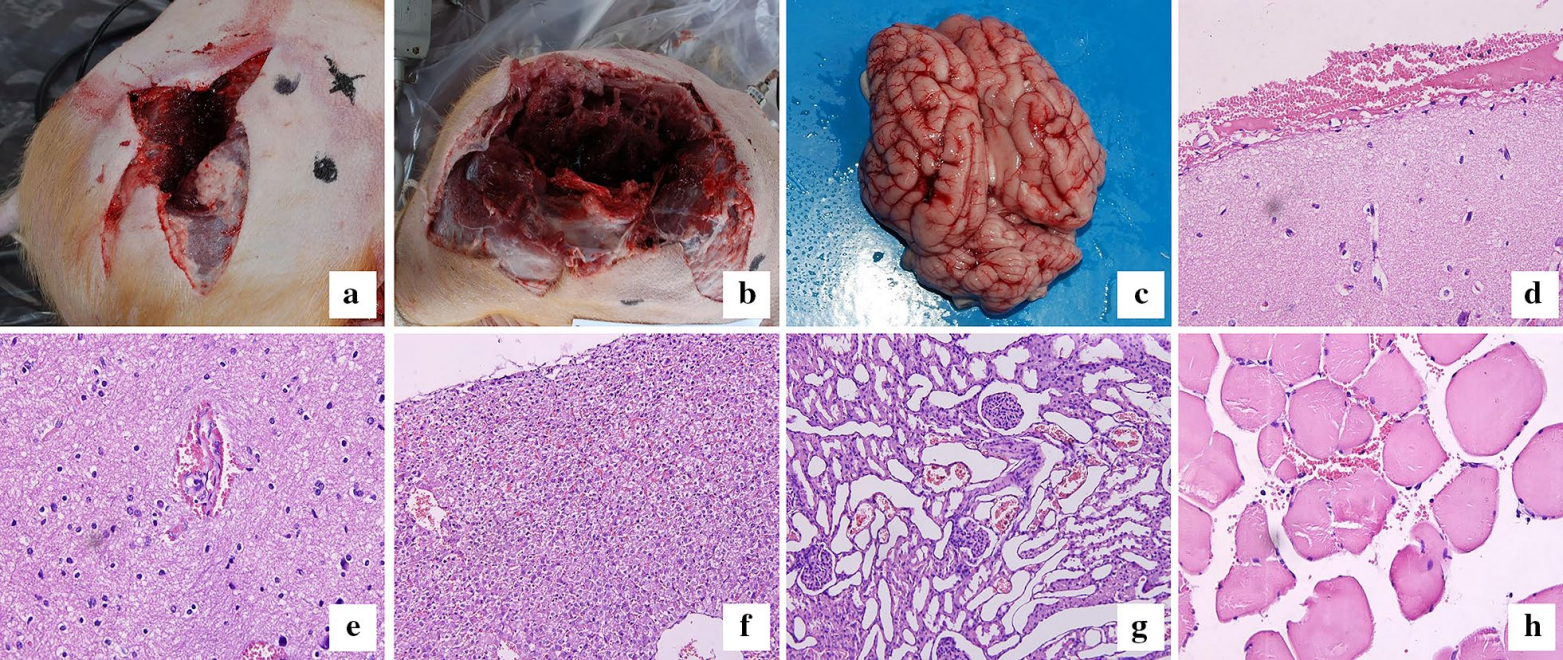
The shape of wound tracts after injury by projectiles with velocities near 3000 m/s (**a**) or 4000 m/s (**b**). Hemorrhage in the cortex of the brain (**c**, **d** hematoxylin and eosin ×20.), microvessel ruptured in the thalamus (**e**, hematoxylin and eosin ×40). Liver in the vicinity of the surface was fragmented and bleeding (**f**, hematoxylin and eosin ×20). Subcortical glomeruli were highly congested and many red blood cells leaked into the tubular spaces (**g**, hematoxylin and eosin ×20). Hemorrhage was observed 7 cm from the surface of the wound tract (**h**, hematoxylin and eosin ×40)

There was an area of pallor around the injury, and outside of the pale area was a blood-flush circle. The pale area increased with the increasing of velocity (Table 1). The entrance was irregular and there was a distinct skin cracking and muscle cavitation when the speed was higher than 2000 m/s (Fig. 1a, b). However, there was only a regular round entrance wound at the speed of 1000 m/s.

In the 1000 m/s group, the wound tract was regular and clear. The injured muscle can be distinguished easily by its color. There was no fracture on the thigh bone. When the projectile velocity exceeded 2000 m/s, the skin and muscle were devastated. Much of the muscle was detached and crisped, and it was very difficult to differentiate the normal tissue from the relatively normal tissue. The thigh bone was broken into pieces, and the tissue was very swollen with large amounts of blood clot.

Judging the muscle according to the 4C principle (color, consistency, contractility, circulation) (Saljo et al. 2003), the range of devitalized muscle was 0.90 ± 0.24, 2.74 ± 0.22, 3.72 ± 0.50, and 4.80 ± 0.51 cm in the 1000, 2000, 3000 and 4000 m/s projectile groups, respectively.

### General pathology of the abdominal and thoracic organs

When the projectile velocity was under 2000 m/s, there were no obvious injuries in the abdominal and thoracic organs. When the velocity reached 3000 m/s, colonic hemorrhage was found. When the velocity was approximately 4000 m/s, the organs were injured severely. The main injuries included colon and bladder hemorrhage, intestinal rupture, lung and liver hemorrhage, and kidney contusion.

### Micropathology of internal organs

There was no obvious hemorrhage in the cortex of brain when the impact velocity was less than 3000 m/s; however, when the velocity reached 4000 m/s, there was obvious hemorrhage in the cerebral cortex (Fig. 1c). Microscopy revealed a vein in the sulcus that had ruptured and caused the subarachnoid hemorrhage (Fig. 1d). As observed under the light microscope, there was a clearly ruptured microvessel in the thalamus after a 3000 m/s impact, and the blood–brain barrier was destroyed, as evidenced by the extravasation of red cells and serum (Fig. 1e).

Although the liver appeared grossly normal with an intact capsule, under the microscope, the parenchyma near the surface was fragmented and bleeding after impacts of greater than 2000 m/s (Fig. 1f). Renal cortical hemorrhage was also found when the projectile velocity was higher than 2000 m/s, and this effect became more severe with increasing projectile velocity. At the speed of 4000 m/s, the subcortical glomeruli were highly congested, with a great number of red cells leaked into the tubular space (Fig. 1g).

An examination of the lung showed that many inflammatory cells and a few red blood cells were leaked into the alveolar space after impacts at the speed of 1000 m/s. However, the volume of red cell leakage increased sharply with increasing projectile velocity. In some cases, the highest velocity impacts caused large areas of lung consolidation.

Bleeding in the muscle of the heart was found from a projectile velocity of 1000 m/s and become more severe with the increasing projectile velocity.

### Micropathology of wound tract

Light microscopy revealed that the range of muscle dissection along the wound tract, as discriminated by necrosis and hemorrhage, is 1.5 cm at the speed of 1000 m/s, 3 cm at the 2000 m/s, 5 cm at 3000 m/s. However, at the speed of 4000 m/s, hemorrhage was observed at a distance of 7 cm from the surface of the wound tract (Fig. 1h).

### Observation by high-speed video camera

**Fig. 2:**
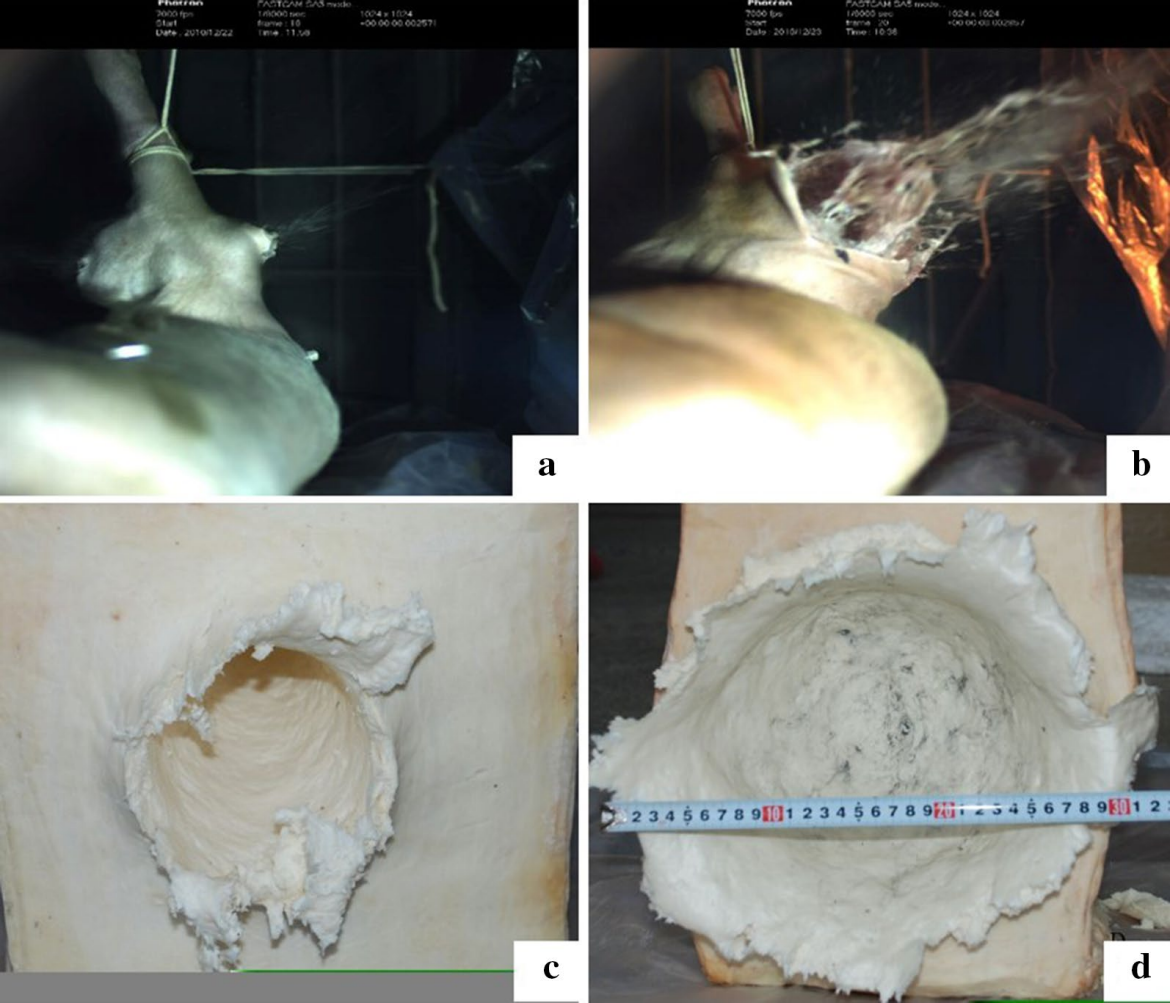
The instantaneous shape of the limb after injury by projectiles whose velocity was approximately 2000 m/s (**a**) or 4000 m/s (**b**). The shape of soap after impact by projectiles whose velocity was approximately 2000 m/s (**c**) or 4000 m/s (**d**)

The high-speed video camera captured the detailed penetrating course of the projectiles. When the speed was lower than 2000 m/s, the projectiles penetrated the limb, and there was little soft tissue splashing (Fig. 2a). When the speed was higher than 3000 m/s, a very large entrance wound was formed with a great amount of muscle splashing (Fig. 2b). The largest diameter of transient cavity was highly related to the projectile speed. The average diameter was 16.43 ± 1.00, 25.83 ± 3.82, 30.00 ± 4.58, and 39.50 ± 6.76 cm when the velocity was 1000, 2000, 3000, and 4000 m/s, respectively (Table 2).

**Table 2:** Pressure detected in the common carotid artery and buttock (n = 5)

Group (m/s)	Diameter of transient cavity^△^ (cm)	Pressure in the artery^▲^ (Kpa)	Pressure in the buttock^※^ (Kpa)
1000	16.43 ± 1.00	27.35 ± 2.79	650.20 ± 165.57
2000	25.83 ± 3.82 b	35.17 ± 5.23 b	1209.40 ± 432.95
3000	30.00 ± 4.58 a	66.31 ± 14.04 a	2011.40 ± 233.77 b
4000	39.50 ± 6.76 a	144.82 ± 35.01 a	3330.00 ± 494.82 a

^△^This diameter was acquired by measured the longest length of the transient cavity in the photo captured by the high speed-camera

^▲^This is the highest positive pressure detected in the common carotid artery

^※^This is the highest positive pressure detected in the buttocks. Compared to the group of 1000 m/s, a < 0.01, b < 0.05

### Shape of soap targets after impact

Special soap targets were used to investigate the transient cavities formed by projectiles with impact velocities of 1000, 2000, and 4000 m/s. The entrance at 1000 m/s was round, with a diameter of 5 cm. The projectiles penetrated the soap, and the exit wound was round, with a diameter of 0.6 cm. When the velocity reached 2000 m/s, the cavity was similar to that observed with a velocity of 1000 m/s; however, the diameter of the entrance and exit wounds were 16 and 1 cm, respectively (Fig. 2c).

However, the projectile did not penetrate the soap and formed a spherical cavity when the impact velocity was approximately 4000 m/s. The diameter was 25 cm wide and 17 cm deep, and under the cavity, some black powder was scattered (Fig. 2d).

### Biomechanics recordings

The distance between the sensors inserted in the buttocks and the target point was 11, 11, 12, and 13 in the 1000, 2000, 3000, and 4000 m/s groups, respectively.

The pressure wave detected from the sensor that was inserted into the buttocks demonstrated several positive and negative peaks. The average highest positive pressure was 650.2 ± 165.57, 1209.4 ± 432.95, 2011.40 ± 233.77, and 3330.00 ± 494.82 kPa at the speeds of 1000, 2000, 3000/s, and 4000 m/s, respectively (Table 2).

The sensor inserted into the common carotid artery recorded several positive peaks, and the average highest positive pressure was 27.35 ± 2.79, 35.17 ± 5.23, 66.31 ± 14.04, and 144.82 ± 35.01 kPa at the speeds of 1000, 2000, 3000, and 4000 m/s, respectively (Table 2).

### Alterations of MBP, NSE, S-100B and IL-6 levels in the serum

The levels of MBP, NSE, and S-100B gradually increased at each post-injury time point (30 min, 1, 3, and 6 h after trauma), compared with the pre-injury time point in the trauma group. However, the levels of these proteins varied unsteadily in the control group. The overall differences in the levels of these three markers were significant between the control and exposed animals (MBP, F = 11.219, *p* = 0.000; NSE, F = 18.844, *p* = 0.000; S-100B, F = 116.505, *p* = 0.000 by RM-ANOVA).

**Fig. 3:**
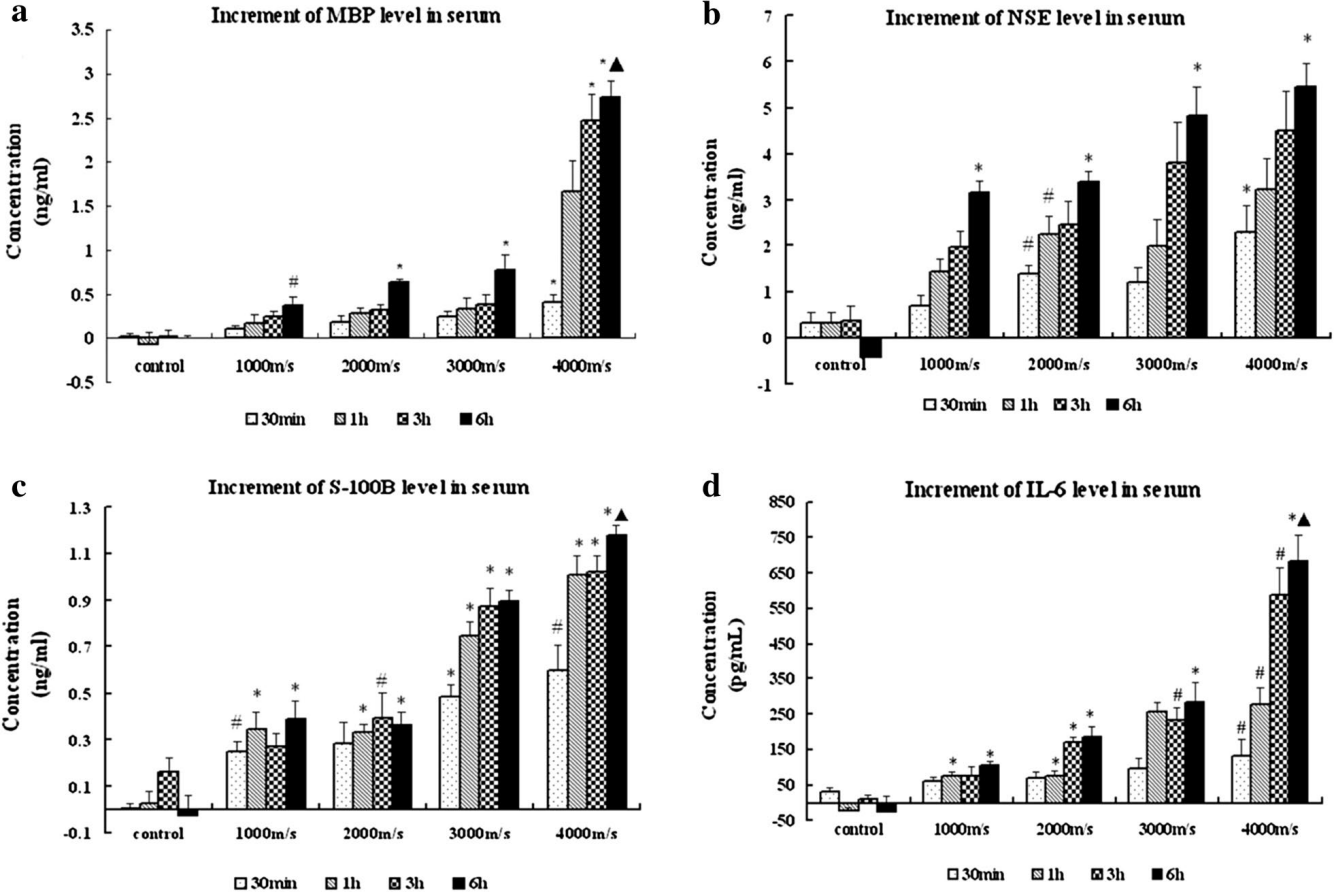
Increment of MBP (**a**), NSE (**b**), S-100B (**c**), and IL-6 (**d**) levels in serum at different times after injury. *p < 0.01 compared to the control group at the same time point after injury.^ #^ *p* < 0.05 compared to the control group at the same time point after injury. ^▲^ *p* < 0.01 compared to the other exposed groups at the same time point after injury

In injured animals, the level of MBP was markedly increased at 6 h (0.37 ± 0.10 ng/ml, *p* = 0.042 in the 1000 m/s group; 0.64 ± 0.03 ng/ml, *p* = 0.001 in the 2000 m/s group; 0.77 ± 0.17 ng/ml, *p* = 0.000 in the 3000 m/s group; 2.73 ± 0.19 ng/ml, *p* = 0.000 in the 4000 m/s group) compared to the control group (−0.002 ± 0.04 ng/ml) after trauma, and the 4000 m/s group was also increased significantly compared with the other injured groups (p = 0.006, 1000 m/s; p = 0.000, 2000 m/s; p = 0.000, 3000 m/s) (Fig. 3a). The level of NSE was markedly increased at 6 h (3.1 ± 0.23 ng/ml, *p* = 0.000 in the 1000 m/s group; 3.38 ± 0.24 ng/ml, *p* = 0.000 in the 2000 m/s group; 4.81 ± 0.65 ng/ml, *p* = 0.000 in the 3000 m/s group; 5.45 ± 0.50 ng/ml, *p* = 0.000 in the 4000 m/s group) compared to the control group (−0.41 ± 0.40 ng/ml) after trauma, and the 4000 m/s group was also increased significantly compared with other injured groups (*p* = 0.003, 1000 m/s; *p* = 0.003, 2000 m/s) (Fig. 3b). The level of S-100B was also markedly increased at 6 h (0.38 ± 0.08 ng/ml, *p* = 0.000 in the 1000 m/s group; 0.36 ± 0.06 ng/ml, *p* = 0.000 in the 2000 m/s group; 0.89 ± 0.05 ng/ml, *p* = 0.000 in the 3000 m/s group; 1.17 ± 0.04 ng/ml, *p* = 0.000 in the 4000 m/s group) compared to 30 min (−0.03 ± 0.08 ng/ml) after trauma, and the 4000 m/s group was also increased significantly compared to the other injured groups (*p* = 0.000, 1000 m/s; *p* = 0.000, 2000 m/s; *p* = 0.006, 3000 m/s) (Fig. 3c).

The level of IL- 6 was also markedly increased at 6 h (184.00 ± 33.29 ng/ml, p = 0.006 in the 2000 m/s group; 285.54 ± 57.58 ng/ml, p = 0.000 in the 3000 m/s group; 683.88 ± 70.94 ng/ml, p = 0.000 in the 4000 m/s group) compared to the control group (−25.05 ± 45.42 ng/ml) after trauma, and the 4000 m/s group was also increased significantly when compared with the other injured groups (*p* = 0.000, 1000 m/s; *p* = 0.000, 2000 m/s; *p* = 0.000, 3000 m/s) (Fig. 3d).

## Discussion

The wounds caused by ballistic projectiles often depend on many factors, such as the impact velocity, the shape and reversion of projectiles, the density of the target, the characteristics of organs, etc. (Santucci and Chang 2004). In our studies, we used spherical steel balls as projectiles, and the results indicated that with increasing velocity, the injury was more severe. At sufficiently high speeds, the impact can cause distant and severe organ damage. The target site pressure, the transient cavity size and the wound entrance size were also highly positively correlated with the projectile velocity.

When the velocity was within the range of 1000–3000 m/s, the projectiles penetrated the hind limb, and the area of entrance and exit were highly positively correlated with the velocity. However, when the speed was approximately 4000 m/s, the projectiles penetrated but did not exit the hind limbs, and caused a very massive entrance wound.

What caused this phenomenon? One theory is that the impact velocity is so high that the projectiles fragmented on impact. In other non-biological hypervelocity impact experiments, it was concluded that when the impact velocity is sufficiently low that the projectiles remain intact, the penetration depth increases with impact velocity, whereas it decreases with the impact velocity when the impact velocity is high enough that the projectiles fragment. At low velocities, the strength of the projectile’s materials is greater than the dynamic impact pressure, and the projectile penetrates the target intact. The crater produced is deep and narrow. With increases in impact velocity, a point is reached at which the impact pressure is sufficient to cause the projectile to fragment into a few large pieces upon impact. Then, as the impact velocity is increased further, the projectile shatters into numerous small pieces and the penetration depth decreases. Finally, a velocity is reached at which the typical fluid impact occurs, and the crater formed is nearly hemispherical in shape. The fragments generated after high-velocity impact are the main reason for the hemispherical cavity because the fragments expand in a direction perpendicular to that of the projectile penetration. When this happens to the living body, it will cause devastating damage to the tissue, as seen in our experiments.

Another theory is shock-induced vaporization. When the velocity is high enough, sufficient energy is released from the high-pressure shocked state that partial vaporization or full vaporization will occur. This is called shock-induced vaporization, and it depends on the materials of the projectiles (Chhabildas et al. 2006). Injury caused by this would be similar to chemical explosives. In our experiments, at the highest impact velocities, the projectile did not fully penetrate the tissue, and caused massive injury. The black powder generated at the bottom of crater in the soap after impact also demonstrated that the steel ball was vaporized upon impact when the velocity was around 4000 m/s.

In our studies, when the velocity is near 1000 m/s, the tissue injury was limited to the wounded area. When the velocity was higher, many internal organs were found to be injured. Especially when the speed neared 4000 m/s, the impact generated a violent shock wave. This wave was similar to an explosive wave and was transmitted throughout the body, causing severe or lethal injuries to many organs. This wave caused hemorrhage and rupture of the hollow organs, and contusion of the solid organs. The results showed that the magnitude of the pressure wave was highly correlated with the velocity when this relationship was tested with identical projectiles.

Changes in brain function and structural damage to the central nervous system (CNS) have been reported following blast injury to the thorax and following penetration of the hind limb with a high-velocity weapon (Wang et al. 2004; Goransson et al. 1988; Suneson et al. 1990; Bauman et al. 2009; Bhattacharjee 2008). This phenomenon has been called a remote or distant effect (Suneson et al. 1987). It was reported that there was no obvious sign of bleeding or tearing of brain tissue and that the blood–brain barrier was destroyed within brain stem and basal ganglia, but rarely in the cortex cerebri when using a 6.0 mm spherical steel ball and an impact velocity of approximately 1500 m/s (Suneson et al. 1987). Perhaps the pressure was not strong enough to tear the vessel. In our experiment, when the impact was approximately 4000 m/s, there was an obvious rupture of vessels in the cortex and parenchyma. This phenomenon further demonstrated that the pressure wave was the main mediator of the distant effects of ballistic impact.

There are several biomarkers used for indicating structural damage to neurons and the supporting cells of the central nervous system, including MBP, NSE, and S-100B (Pouw et al. 2009). When the blood–brain barrier is damaged, these proteins gain access to the peripheral circulation (Begaz et al. 2006). The results showed that there was a different type of injury to the nerve cells when the hind limb was injured by high-velocity projectiles and that the extent of injury was correlated with the velocity when the projectiles were the same.

After impact by the high-velocity projectiles, the local and distant organs were injured severely, which could lead to systemic inflammation. IL-6 is used as an indicator of inflammatory status and is useful in the evaluation of the clinical status of trauma patients. It was demonstrated that IL-6 concentrations after trauma were associated with injury severity and adverse outcomes, including multiorgan dysfunction (MOD) and death (Giannoudis et al. 2008; Jawa et al. 2011). There is also evidence that cytokines of the IL-6 family translocate across the damaged blood–brain barrier after traumatic brain injury and may play an important role in the subsequent development of multiorgan failure (Jawa et al. 2011).

Our results also demonstrated that injury extent was highly correlated with the IL-6 serum level. With increased impact velocity, the injury was more severe, and the level of IL-6 was greatly increased. The extremely high level of IL-6 observed demonstrated that there was a severe inflammatory reaction after the super-high-velocity impact on the body.

Penetrating wounds depend on many factors, such as the shape and composition of the projectile (Saljo et al. 2003). However, when identical projectiles are used, the results in our experiment showed that the wound depends on the projectile velocity. Different velocities cause different patterns and amounts of destruction of the target tissue. When the velocity was less than 1000 m/s, the impact only destroyed some muscle and did not impact the bone.

When the velocity was higher than 2000 m/s, the powerful pressure wave caused serious muscle destruction and caused a comminuted fracture of the thigh bone, which could lead to a second injury. Even indirect fracture caused by ballistic impact of velocity less than 1000 m/s have been reported (Kiser et al. 2013). When the velocity was approximately 4000 m/s, the impact caused the hind limb devastating damage and made an enormous soft tissue and skin defect. If this impact occurred in the abdominal or chest region, it could cause sudden death, not to mention impact in the head.

Not only the local tissue but also the organs far from the wound should be monitored in such a case. MOD was prone to occur when the super-high velocity projectiles were used. It was reported that severe blast injury could overwhelm the host immune function and cause systemic inflammatory reactions (Hawksworth et al. 2009; Cobb et al. 2000). The results of super-high impact were very similar to blast injury.

Although our experiments have defined the characteristics of wounds after different super-high velocity impacts on the limbs, functional studies and special biomarkers for the different vital organs need to be performed for clinical purposes. Furthermore, we do not yet know what the injury profile would be with projectile velocities much higher than 4000 m/s.

## Conclusion

The extent of injury was correlated with the velocity when the impact occurred under the terminal velocity, and when the projectile used was identical. There is a terminal velocity for each projectile, which depends on the different projectile materials and on the atmosphere. At the terminal velocity, the impact would make the projectile melt or vaporize and release massive amounts of energy that could cause severe damage.
